# Tunable Doping and Optoelectronic Modulation in Graphene-Covered 4H-SiC Surfaces

**DOI:** 10.1021/acs.jpcc.4c06409

**Published:** 2025-02-14

**Authors:** Masoud Mansouri, Fernando Martín, Cristina Díaz

**Affiliations:** † Departamento de Química, Módulo 13, 16722 Universidad Autónoma de Madrid, Madrid 28049, Spain; ‡ Instituto Madrileño de Estudios Avanzados en Nanociencia (IMDEA Nano), Campus de Cantoblanco, Madrid 28049, Spain; § Departamento de Química Física, Facultad de CC Químicas, Universidad Complutense de Madrid, Madrid 28040, Spain

## Abstract

Semiconducting graphene is pivotal for the advancement of nanoelectronics due to its unique electronic properties. In this context, silicon carbide (SiC) surfaces have been proposed as ideal supports for inducing semiconducting characteristics in graphene. Here, we employ many-body perturbation theory to investigate the electronic structure and optical properties of graphene-covered 4H-SiC surfaces. Our analysis reveals that pristine 4H-SiC surfaces with dangling bonds exhibit a reduced transport gap and enhanced optically active states within the visible spectrum compared to bulk 4H-SiC. Strong interfacial interactions resulting from the adsorption of a single graphene layer (GL) significantly alter graphene’s dispersion, yielding a semiconducting interface with modified optoelectronic properties. While the addition of a second GL restores Dirac dispersion, the two polar faces of the underlying 4H-SiC induce either metallic n-type doping or behavior similar to that of freestanding graphene. Furthermore, we investigate the adsorption of a molecular electron acceptor on SiC covered with one and two GLs. Our findings reveal notable renormalization of the molecular energy levels upon adsorption, resulting in the emergence of distinct new optically excited states. Additionally, a shift in the Fermi level, attributed to partial charge transfer, indicates effective p-type doping. The tunable doping characteristics and optical profiles across various energy ranges highlight the potential of graphene-covered 4H-SiC surfaces as versatile materials for a wide range of technological applications.

## Graphical abstract

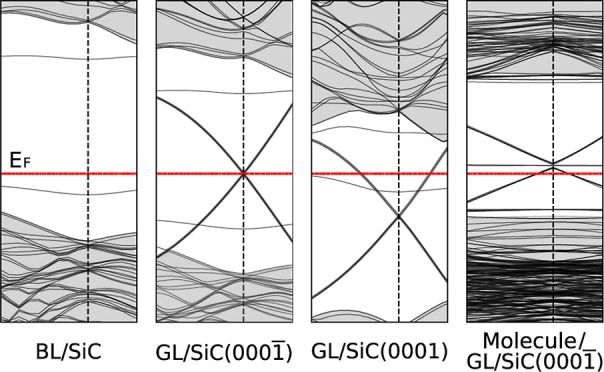

## Introduction

Polytypes of silicon carbide (SiC) have garnered significant attention from the scientific community due to their unique properties and versatility. Among various intrinsic applications,^1^ SiC surfaces have been proposed as ideal substrates for the growth of graphene layers (GLs).^2–13^ Notably, the formation of GLs on different SiC polytype surfaces is highly dependent on the underlying structure.^6,12^ For instance, the epitaxial GLs on hexagonal polar,^7–11^ nonpolar,^14,15^ and cubic surfaces^16,17^ exhibit notably different characteristics, caused by surface charge transfer.^3–5,7–11^ Consequently, different configurations of GL/SiC interfaces have been proposed for vast numbers of technological applications.^6–11,13^

In this work, we investigate the hexagonal 4H-SiC surface, focusing on its two polar faces: the C-terminated (0001̅) and the Si-terminated (0001). These surfaces are commonly used in the fabrication of semiconductor devices and multilayer heterostructures.^7–11^ The growth of epitaxial GLs on these substrates begins with the formation of a graphene-like buffer layer (BL), which is covalently bonded to the underlying SiC substrate and lacks the semimetallic dispersion of freestanding graphene.^5,7,9,18,19^ In contrast, adsorption of the subsequent GLs restores the pristine graphene features, though with modified electronic properties, including shifts in their work functions, due to interfacial charge transfer, polarization, and doping effects. In parallel with these experimental observations, standard density functional theory (DFT) has been widely used to identify the corresponding electron energy spectra and the nature of the interactions at the interface: strong interactions for the BL/SiC interface and weak van der Waals (vdW) interactions for the subsequent GLs.^9,12,20^

Considering the potential applications of graphene-covered SiC surfaces in optoelectronics, accurate descriptions of their electronic structure and optical absorption spectra are still relatively rare, with most studies focusing on the bulk and clean surfaces of SiC polytypes.^21–24^ Here, we employ many-body perturbation theory to thoroughly investigate the optoelectronic properties of both polar faces of 4H-SiC, with and without GLs. Given the complex orbital-dependent properties of hexagonal SiC crystals,^22^ it has been demonstrated that *GW* corrections^25^ are crucial for achieving accurate results, thanks to the dynamic electronic correlation accounted for in the method.^26^ To obtain reliable optical absorption spectra, we further incorporate bound excitonic effects by solving the Bethe–Salpeter equation (BSE).^26^ This combined *GW*/BSE approach allows us to accurately describe both charged and neutral excitations, required for the quantitative description of quasiparticle and absorption spectra, often showing excellent agreement with experimental results.^21,23,24,26,27^ Additionally, we employ DFT and its time-dependent extension using the hybrid Heyd–Scuseria–Ernzerhof (HSE) functional,^28^ which incorporates screened Coulomb interactions and a fraction of exact exchange for short-range interactions. Although the HSE functional provides a more accurate description of the electronic bandgap than purely (semi)­local functionals, it is known to struggle with capturing long-range electron–hole (e–h) interactions, thereby limiting its ability to characterize bound excitons accurately.^29–31^ Despite this limitation, the HSE functional can still produce reasonable continuum spectra.^30^

Charge-transfer driven by molecular adsorption on graphene has been widely recognized as a promising method for compensating excess negative charges in epitaxial GLs.^13,32,33^ Likewise, molecular adsorption on the GL/SiC interfaces could potentially modify the electronic structure, offering valuable insights into the rearrangement of the electron density and possible charge transfer. Building on recent studies,^13,24^ we investigate the adsorption of the electron-accepting molecule F6TCNNQ (2,2′-perfluoro-naphthalene-2,6-diylidene dimalononitrile, also known as F6-TNAP^34^) onto GL/SiC interfaces. Our focus is on the SiC(0001̅) underlying surface, for which the formation of high-quality GLs with larger domain sizes and improved charge carrier mobility has been observed.^7,11,24^ As we demonstrate, a suitable bandgap size upon adsorption of the BL, and pristine-like (neutral) characteristic of the ensuing GLs, make the C-terminated substrate an excellent candidate to fine-tune the optoelectronic properties of GL/SiC interfaces through the adsorption of molecules whose substrate-dependent energy levels can introduce trap or recombination centers within the substrate.

This article begins with a brief review of the computational methods, followed by a comprehensive analysis of the quasiparticle and absorption spectra of the two polar faces of the clean 4H-SiC surface. Next, we evaluate the impact of adsorbing one and two GLs onto the 4H-SiC surfaces, highlighting key features in the band structures arising from interfacial interactions, charge transfer, and doping effects. Our results reveal that while BL/SiC interfaces on both polar faces retain the semiconducting nature of 4H-SiC, albeit with a reduced band gap, the presence of surface states and interface interactions significantly modulate the optical properties. Furthermore, we demonstrate that the adsorption of a second GL leads to a metallic interface with n-type doping characteristics on the Si-terminated face and properties resembling freestanding graphene on the C-terminated face. Finally, we investigate the adsorption of the F6TCNNQ molecule on the GL/SiC interfaces, revealing a substantial renormalization of the adsorbate energy levels due to surface-induced screening. This renormalization results in distinct optically excited states arising from the hybridization of molecular and substrate states, along with p-type doping. The tunable p- or n-type doping characteristics of graphene-covered SiC surfaces make them highly versatile for advanced applications in optoelectronics and interface engineering.

## Computational Details

Our primary substrate is hexagonal 4H-SiC, modeled using the experimental lattice constants.^24,35^ GLs grown on C-terminated 4H-SiC exhibit a complex periodicity of $\sqrt{3}\times \sqrt{3}$ R30° (abbreviated as $\sqrt{3}$ R),^8–10,36^ while on the Si-terminated face, the larger $6\sqrt{3}\times 6\sqrt{3}$ R30° reconstruction has been observed.^2,12^ Although this latter configuration provides stress-free alignment at the Si-terminated interface, the large number of atoms in the primitive cell makes computational studies costly, even at the mean-field level.^37^ To facilitate higher-level theoretical studies while keeping computations tractable, we employed the same $\sqrt{3}$ R reconstruction commensurated with four graphene unit cells for both polarities of the 4H-SiC surface, along with a vacuum interval of 14 Å, as shown in Supporting Information Figure S1. This simplified interface model, implying an elongation in the atop GL, is widely regarded as a practical approximation with no significant modification in the electronic structure compared to that of freestanding graphene.^9,12^

To investigate the adsorption of the F6TCNNQ acceptor, we focus on the C-terminated 4H-SiC covered by GL(s). Given the size of the F6TCNNQ molecule, our adsorption energy studies indicate that a 3 × 3 primitive cell of the $\sqrt{3}$ R reconstruction is necessary to minimize interactions among periodic images of the adsorbate. Furthermore, the DFT-HSE results presented in Supporting Information Figure S2 confirm that variations in the bandgap and near-edge band structures are minimal across models with three to five SiC bilayers.^24^ Therefore, a 3 × 3 three-SiC-bilayer supercell model was used to mitigate the computational cost of molecular adsorption. It is worth noting that the Dirac cone of freestanding graphene appears at the Γ-point when their primitive cell is 3*N* × 3*N* (*N* is an integer).^38,39^

The geometry optimization of all studied systems was carried out using spin-polarized DFT-HSE, as implemented in the Vasp code.^40^ We applied projector augmented wave potentials and the Grimme correction^41^ for vdW interactions. A plane wave cutoff energy of 400 eV and a Γ-centered 8 × 8 × 1 *k*-point grid were used, ensuring total energy convergence and interatomic forces were below 10^–5^ eV and 5 meV/Å, respectively. For substrates with the F6TCNNQ adsorbate, the HSE equilibrium geometry was obtained using single Γ-point sampling and a force convergence criterion of 0.01 eV/Å, while the actual HSE calculations were performed using a 2 × 2 × 1 *k*-point grid. All HSE band structures were explicitly computed along a specified *k*-path given by ref 42.

To compute the *GW* quasiparticle corrections, we used the perturbative one-shot *G*_0_ *W*_0_ approach.^26^ This involved constructing noninteracting Green’s functions (*G*_0_) based on mean-field Perdew–Burke–Ernzerhof (PBE) and HSE solutions, with the dynamical screened interaction (*W*_0_) obtained at the random phase approximation (RPA) level.^26^ For pristine and graphene-covered surfaces, *G*_0_ *W*_0_ calculations were conducted using Vasp with an 8 × 8 × 1 mesh sampling and a total of 1240 bands, provided a convergence of 0.1 eV in the direct bandgaps. The electronic band structures along the specified *k*-path were obtained by interpolating the initial quasiparticle energies using localized Wannier functions.^43^ Optical absorption and excitonic properties were subsequently calculated by solving the BSE within the Tamm–Dancoff approximation (TDA),^26^ considering 12 bands on either side of the Fermi level. The same number of e–h states was used for solving the Casida equation^26^ to obtain time-dependent DFT-HSE (TDHSE) spectra.

The *GW* calculations for the surface involving F6TCNNQ were performed using the BerkeleyGW package,^44^ with DFT-PBE input generated by Quantum Espresso.^45^ Norm-conserving pseudopotentials with a kinetic energy cutoff of 120 Ry were applied. The frequency dependence of the dielectric function was modeled using the plasmon-pole approximation,^26^ with a 2 × 2 × 1 sampling grid and a cutoff energy of 14 Ry. A total of 3000 bands were included, covering approximately 30 eV above the Fermi energy. The BSE-TDA spectrum was calculated using 14 occupied and 18 empty bands, with the BSE kernel interpolated to a finer Brillouin zone sampling of 8 × 8 × 1.

The quasiparticle and optical properties of isolated F6TCNNQ were calculated using the all-electron Molgw code^46^ with a cc-pVQζ basis set. Given the lower computational cost in the gas phase, quasiparticle energies were computed using the *G*_0_ *W*_0_ and the eigenvalue-only self-consistent *GW* (ev*GW*) schemes. Note that the quasiparticle energies of the lowest unoccupied molecular orbital (LUMO) and highest occupied molecular orbital (HOMO) of an isolated molecule can be directly linked to the negative electron affinity (EA) and ionization energy (IE), respectively.^26,47^ Optical absorption spectra, averaged over the three Cartesian components, were obtained by diagonalizing the BSE-TDA solutions.

## Results & Discussion

### Pristine 4H-SiC Surfaces

We begin our study by investigating the clean surfaces. The reconstructed 4H-SiC surfaces were modeled with five SiC bilayers, including one more periodic bilayer than the bulk unit cell. This configuration ensures that the topmost and lowest SiC bilayers are identical, preserving the same stacking sequence in the underlying structure for both termination faces.

In the C-terminated surface model (0001̅), one face consists of three unpaired C atoms, as illustrated in Figure 1a, while the Si atoms on the opposite side are H-passivated. HSE-relaxed geometry indicates that the unpaired C atoms in the topmost layer have a bond angle of 114° with the adjacent Si atoms, which is approximately 4% larger than the corresponding angles among Si–C–Si atoms in the lower layers. This increase results in a vertical distance of 0.42 Å (2.35 Å) between the topmost C layer and the lower Si (C) layer, which is 0.15 Å less than the distance in other layers.

**Figure 1:**
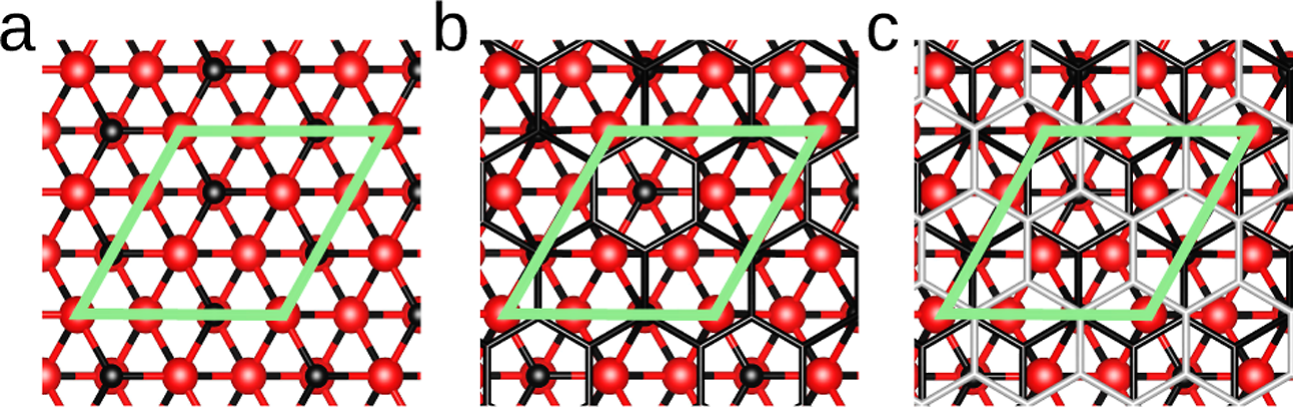
Top view of (a) the 4H-SiC(0001̅) surface, (b) covered by the BL, and (c) the subsequent GL. The green hexagon outlines the $\sqrt{3}$ R primitive cell. Red and black balls represent Si and C atoms. The black and silver sticks illustrate the BL and the subsequent GL, respectively.

The three topmost C atoms with dangling bonds in the 4H-SiC(0001̅) surface impose three singly occupied states with a dominant role of p_*z*_ states in the band structures, as depicted in Figure 2a. Compared to the bulk phase, which has an indirect gap of 3.29 eV and a direct gap of 4.56 eV (6.16 eV) at the M-point (Γ-point),^24^ the presence of these in-gap states leads to notable changes in the electronic structure of the 4H-SiC(0001̅) surface. Particularly, this surface features a spin-down direct gap at the Γ-point, with values listed in Table 1 for three theoretical approaches. As previously suggested,^21^ the semiconducting ground state points toward the occurrence of a Mott–Hubbard transition associated with a splitting corresponding to the on-site Coulomb repulsion energy. Our results similarly reveal a sizable splitting among the dangling-bond-induced states; while the HSE and *G*_0_ *W*_0_@PBE methods suggest a mean splitting of 1.7 eV, the *G*_0_ *W*_0_@HSE approach predicts a 0.5 eV larger value. Moreover, the dispersion of these in-gap states at the HSE level shows an increase of 0.5 eV (0.2 eV) for the occupied (empty) bands compared to those of the PBE. By applying the quasiparticle correction, this dispersion has been slightly increased, particularly for the empty states, for which appropriately accounting for the correlation energies is crucial.

**Figure 2:**
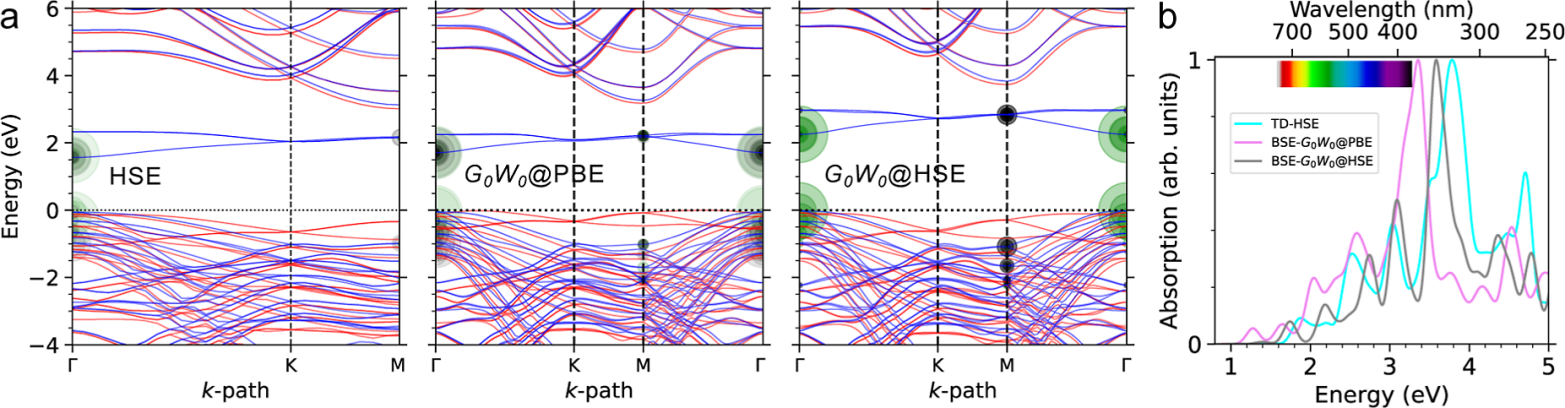
(a) The electronic band structures of 4H-SiC(0001̅) obtained using three theoretical approaches. Spin majority and minority are represented in red and blue, respectively, with the valence band maximum (VBM) set to zero energy. (b) The corresponding optical absorption spectra, broadened using a Gaussian factor of 0.08 eV. The e–h eigenvalues involved in the excitations at the first two peaks are depicted by green and black circles, with magnified radii for clarity, representing the absolute value of the coupling coefficients.

**Table 1:** Work Functions (Φ) and Direct Bandgap Energies at the Γ and M Points, Calculated for the Majority (Minority) Spin Channel Using Different Theoretical Approaches^a^

	Φ	*k*-point	*G*_0_*W*_0_@PBE	HSE	*G*_0_*W*_0_@HSE
4H-SiC(0001̅)	6.28	Γ	4.81 (1.68)	4.84 (1.62)	5.43 (2.27)
		M	3.21 (3.32)	3.34 (3.15)	4.02 (3.98)
BL/4H-SiC(0001̅)	6.04	Γ	3.34 (2.57)	3.19 (2.71)	3.87 (3.19)
		M	2.64 (3.45)	2.73 (3.52)	3.22 (4.08)
4H-SiC(0001)	5.23	Γ	3.49 (3.12)	3.32 (3.20)	3.82 (3.68)
		M	2.26 (3.85)	1.87 (3.85)	2.79 (4.41)
BL/4H-SiC(0001)	3.56	Γ		0.42 (2.98)	1.09 (3.63)
		M		0.76 (2.86)	1.35 (3.46)

a The Φ values were obtained at the DFT-HSE level. All values are in eV.

For the reconstructed 4H-SiC(0001̅) surface, the optical absorption spectra are shown in Figure 2b. Compared to the bulk, which exhibits an absorption onset in the ultraviolet (UV) region,^1,22–24^ the C-terminated surface displays a pronounced red-shift and a broadened absorption spectrum that extends into the visible energy range. From a theoretical perspective, it is noteworthy that the optical structures predicted by TDHSE and BSE-*G*_0_ *W*_0_@HSE are in close agreement. The BSE-*G*_0_ *W*_0_@HSE calculations predict an absorption onset at 1.39 eV, corresponding to a substantial exciton binding energy (ϵ_b_) of 880 meV, followed by prominent peaks at 1.7, 2.2, 2.7, and 3.1 eV within the visible range. In comparison, TDHSE calculations estimate a slightly blue-shifted optical onset at 1.59 eV, reflecting a much smaller ϵ_b_ of 30 meV, with strong transitions at 1.8, 2.2, 2.5, and 3 eV. Both methods reveal qualitatively similar characteristics for these transitions. Specifically, for the two lowest-energy peaks, the e–h contributions with the largest coupling coefficients, illustrated by the circles in Figure 2a, predominantly reside in the spin-down channel and correspond to transitions from hole states at the bulk-like SiC valence edge to unoccupied in-gap surface states. The BSE-*G*_0_ *W*_0_@PBE spectrum, while qualitatively similar to those obtained using TDHSE and BSE-*G*_0_ *W*_0_@HSE, exhibits a red-shift, with the first two peaks appearing in the near-infrared (NIR) region. Despite this shift, the e–h contributions remain consistent with those identified in the other two approaches. Therefore, it implies that this red-shift arises mostly from differences in quasiparticle bandgaps and points to the well-known starting-point dependency within the one-shot *GW* approach.^47^

The other polar face, 4H-SiC(0001), was modeled with the same bilayer stacking order as the C-terminated face, including three unpaired Si atoms on one side and H-saturated carbon atoms on the opposite side (see Figure S1 in Supporting Information). Equilibrium HSE geometry for this model reveals minor changes of approximately 1% in topmost C–Si–C bond angles, resulting in an almost negligible variation in the interlayer distances. Figure 3 presents the electronic structure and optical characteristics of this Si-terminated surface. The band structures, calculated using different methods, consistently show the presence of three singly occupied in-gap states. These surface states, stemming from the dangling bonds of the topmost Si atoms, exhibit slightly larger splitting than seen in the C-terminated surface in Figure 2a. Additionally, the Si-terminated surface states are separated by 0.5–0.7 eV from the bulk states in the valence manifold, aligning with the photoelectron measurements.^12^ This separation can be linked to the delocalization of Si dangling bonds and their higher energy compared to C atoms, which results in an upward shift of the Fermi energy and a reduced work function as given in Table 1. Consequently, the band structures across the three theoretical levels indicate that the 4H-SiC(0001) surface possesses larger direct gaps than its C-face counterpart (see Table 1). These larger direct gaps contribute to a blue-shifted optical spectrum for 4H-SiC(0001), with the main peak around 4 eV and additional visible absorption peaks at about 2.6 and 3.2 eV, as shown in Figure 3b. The e–h states responsible for the peaks within the visible spectrum involve contributions from the valence bulk-like states to the surface states (see circles in the band structures). Additionally, all spectra include a set of weakly allowed transitions between 1.8 and 2 eV, corresponding to transitions between spin-majority surface states and the M-point conduction edge.

**Figure 3:**
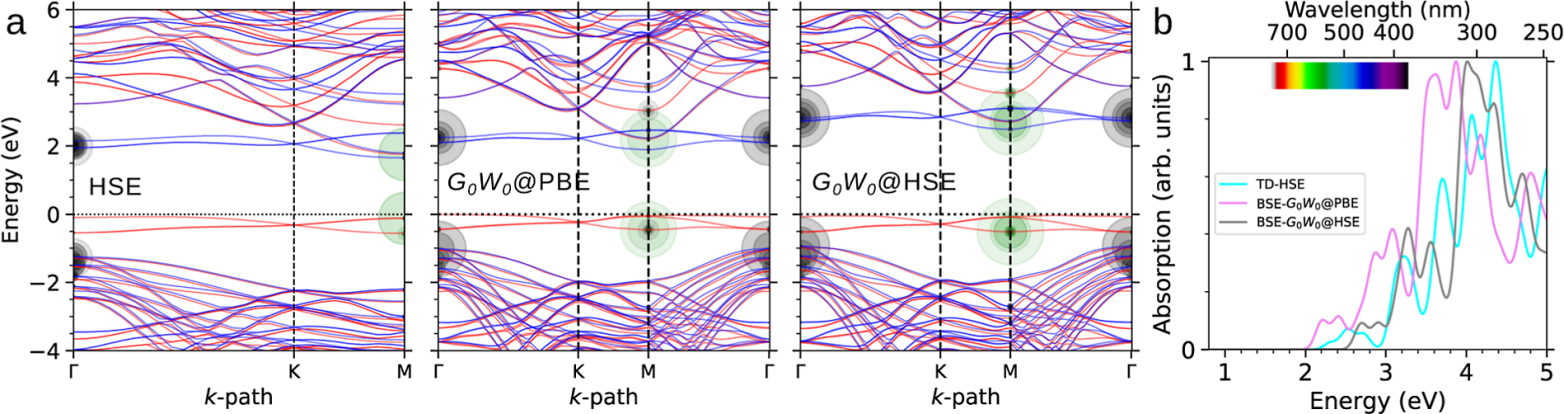
(a) The electronic band structures and (b) the optical absorption spectrum of 4H-SiC(0001).

### Graphene Adlayer on the 4H-SiC Surfaces

The most energetically stable stacking order of GL/4H-SiC(0001̅) is illustrated in Figure 1b. At the interface, two C atoms of the surface are covalently bonded to the overlying GL, while the third atom, referred to as the C-deficient atom (C_D_), is positioned at the hollow site of the graphene ring and remains with a dangling bond. The HSE geometry optimization indicates a bond length of 1.62 Å among bonded C atoms at the interface, in excellent agreement with experimental measurements,^8^ while the C_D_ atom relaxes outward. This results in a corrugation of 0.4 Å on either side of the interface, with a mean separation of 2.4 Å between the GL and the substrate. The stacking of the GL on the Si-terminated face follows a similar pattern: at the interface, the Si atom beneath the graphene hollow site, known as the lonely Si (Si_L_), includes one unpaired electron per unit cell, while the other two Si atoms form direct bonds with graphene, with a bond length of 2 Å, which is 6% longer than the corresponding bonds in the lower layers (see Figure S1 in Supporting Information). Consequently, the equilibrium geometry shows a corrugation of 0.3 Å at the interface. The formation of covalent bonds at both C- and Si-terminated interfaces, combined with the distorted geometry, suppresses key features of free-standing graphene and causes the graphene adlayer to behave as a BL.^7,12,24^

Figure 4a shows the electronic band structure of 4H-SiC (0001̅) covered by the BL obtained with three different methods. Notably, the presence of a singly occupied state, induced by the p_*z*_ state of the C_D_ atom, along with BL bands at the edges (see thicker lines in the HSE band structure), maintains the insulating phase of the substrate. Despite variations in bandgaps across different theoretical approaches, as listed in Table 1, we confirmed that the spatial distributions of the associated orbitals are comparable. For the C_D_-induced state, moreover, all band structures reveal a strongly localized characteristic with a small dispersion of 0.1 eV and a mean splitting of 2, 2.5, and 2.9 eV at the *G*_0_ *W*_0_@PBE, HSE, and *G*_0_ *W*_0_@HSE levels, respectively. Compared to the clean surface in Figure 2, the smaller band dispersion of the C_D_-induced state can be attributed to the passivation effect imposed by the BL, which reduces the influence of the SiC substrate on the unbound atom, resulting in stronger localization of the state.

**Figure 4:**
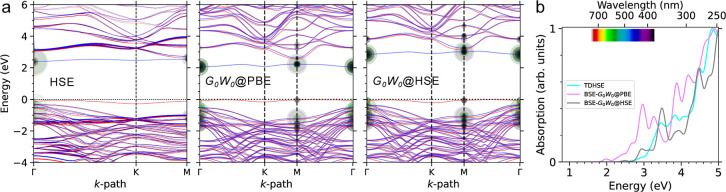
(a) The electronic structure and (b) optical properties of the BL/4H-SiC(0001̅). In the HSE band structures, the contribution of the BL is highlighted with thicker lines.

The optical absorption spectrum of the BL/4H-SiC (0001̅) is shown in Figure 4b. Similar to the previous cases, TDHSE and BSE-*G*_0_ *W*_0_@HSE spectra are in close agreement, suggesting a main peak at 5 eV and a clear trace in the visible energy window, followed by two strong transitions at about 2.9 and 3.4 eV. Examining the involved e–h states, we found that these two excitations mainly stem from transitions between the spin-down valence bands and the unoccupied C_D_ state, as visualized by green and black circles on the band structures. The 3.4 eV transition additionally features some excitations between holes at the occupied C_D_ state and electrons at near-edge BL states. The BSE-*G*_0_ *W*_0_@HSE and TDHSE spectra also feature weakly allowed transitions at 2.4 and 2.7 eV, respectively, corresponding to the transitions between spin-majority edges. The BSE-*G*_0_ *W*_0_@PBE spectrum, on the other hand, qualitatively aligns with the two other spectra, with a systematic red-shift of 0.5 eV over the visible window, linked mostly to gap differences.^26,47^

Now we focus on the electronic structure of the Si-terminated 4H-SiC surface covered by a BL. Like previous calculations,^9^ DFT-PBE calculations for this system predict a half-metallic ground state, with an upward-shifted Fermi level pinned by the Si_L_ state near the conduction edge (see Supporting Information Figure S3). However, calculations using the hybrid HSE functional, shown in Figure 5a, reveal a semiconducting ground state with separated Si_L_ states on either side of the Fermi. The discrepancy between the PBE and HSE estimations can be attributed to the limitations of the semilocal PBE functional, known to underestimate electronic correlations and misrepresent exchange interactions, particularly in systems with strong electronic localization or correlation effects. Consequently, we place greater confidence in the HSE-derived semiconducting ground state, which indicates a spin-majority direct transport gap of 0.42 eV. This is further corroborated by *G*_0_ *W*_0_ quasiparticle corrections, which refine the energy levels and yield a direct Γ-point gap of 1.09 eV (see Table 1).

**Figure 5:**
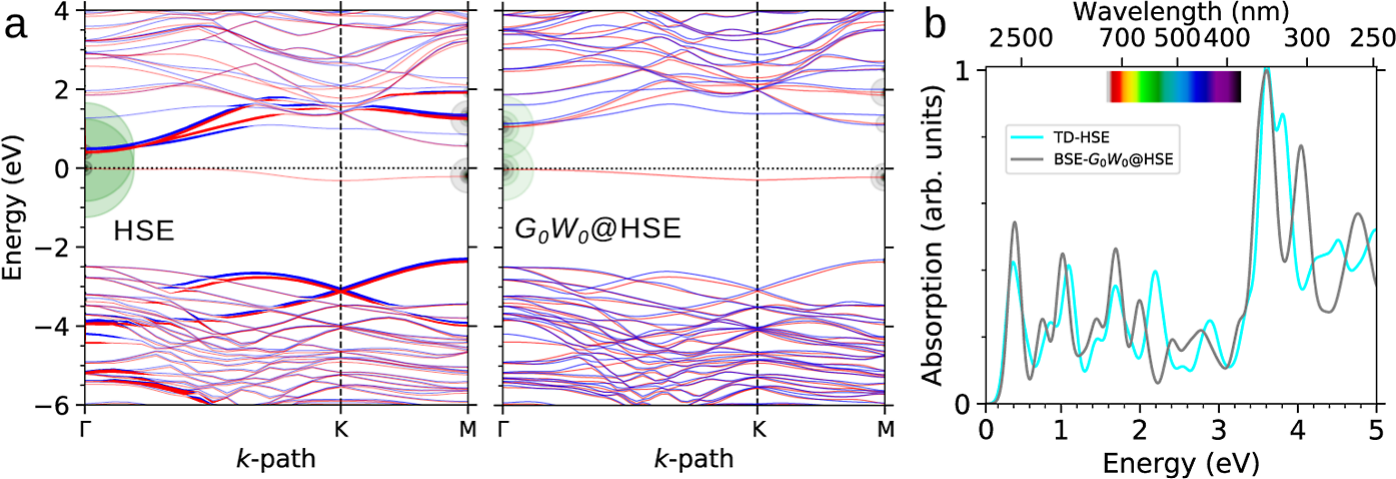
Electronic structure and optical properties of BL/4H-SiC(0001).

Due to the delocalization of the Si surface states, as discussed for the corresponding clean surface, the dangling-bond-induced states exhibit greater dispersion with smaller splitting compared to the C_D_ state in Figure 4. A comparison among the bulk states in the band structures of Figures 4 and 5, moreover, shows the shift in the Fermi level (and the consequent impact on the work function) on the Si-terminated face compared to the C-terminated face. This indeed indicates strong interactions at the interface between the Si-terminated face and the BL, leading to the charge transfer and n-type doping in the BL/SiC(0001). As a result, Bader charge analysis indicates that the BL on the Si-terminated face gains an excess charge, primarily accumulated on the atoms bonded to the two Si atoms at the interface, while the BL on the C-terminated face remains nearly neutral.

The optical absorption spectrum of BL/SiC(0001) is depicted in Figure 5b. Despite the differences in bandgap values estimated by HSE and *G*_0_ *W*_0_@HSE, the remarkable agreement between their corresponding optical structures highlights the significant ϵ_b_ of 0.6 eV in the BSE solution. Both theoretical spectra exhibit a broad optical absorption range with a prominent mid-infrared excitation at approximately 0.4 eV (3100 nm), along with additional peaks at 0.76 and 1 eV. The e–h coupling coefficients of these excited states, represented by circles in the corresponding band structure, indicate transitions from the singly occupied Si_L_ state to the conduction band edge within the spin-majority channel. Importantly, the bulk-like hole states contribute dominantly to the absorption spectra at around 2.86 eV, closely resembling the absorption profile of the pristine surface.

### BL/4H-SiC Covered with an Additional GL

The adsorption of an additional GL in the Bernal stacking configuration is identified as the most energetically favorable arrangement.^3,9,12^ Analyzing the HSE equilibrium geometry for both polar faces reveals that adding a second GL leads to negligible changes in the underlying structure. This is indeed consistent with the nearly planar structure of the second GL, which maintains a mean distance of 3.32 and 3.46 Å from the BL on Si- and C-terminated 4H-SiC, respectively. For comparison, the experimental value for the graphite interlayer distance (at room temperature) is determined to be 3.4 Å (3.6 Å).^3,37^ Consequently, the vdW bonding between the GL and the underlying BL/4H-SiC is expected to preserve the intrinsic graphene dispersion.

The HSE band structure of GL/BL/4H-SiC (0001̅) in Figure 6a confirms the appearance of the C_D_ states on either side of the Fermi level alongside the graphene-like dispersion. Similar to freestanding graphene, the C_*p*_ orbitals in the topmost GL evolve into delocalized π–π* states at the band edges, resulting in the Dirac crossing-point and semimetallic characteristics. It is also worth noting that the *GW* corrections applied on top of PBE and HSE yield a similar zero-gap band structure, as shown in Supporting Information Figure S4, with a more pronounced dispersive nature and slight variations in the C_D_ energies.

**Figure 6:**
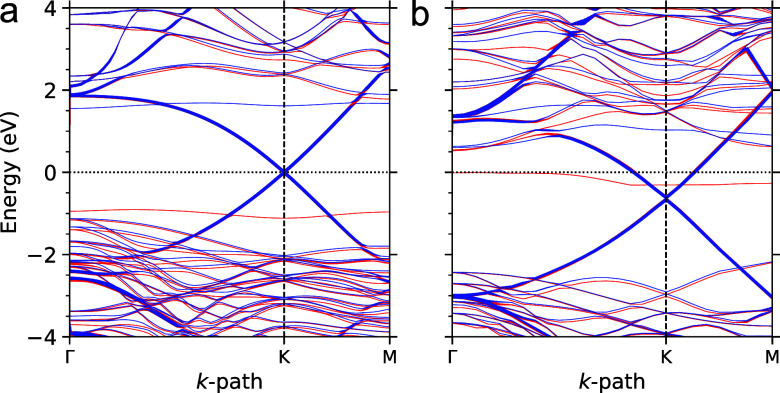
HSE band structure of the (a) C-terminated and (b) Si-terminated faces of the $\sqrt{3}$ R 4H-SiC covered by two GLs. Thicker lines highlight the contribution of the topmost GL.

The adsorption of the subsequent GL on the BL/4H-SiC(0001), as shown in Figure 6b, also exhibits a graphene-like dispersion intersected by Si_L_ states. However, HSE calculations indicate that the Dirac point is shifted 0.6 eV below the Fermi energy, signaling an n-type doping in the topmost GL. This result is consistent with recent UV-excited ARPES measurements,^13^ which point to intrinsic n-type doping with the Fermi energy positioned approximately 0.42 eV above the Dirac point. This indeed reflects a charge-transfer process between the BL/4H-SiC(0001) substrate and the ensuing GL, further highlighting the interfacial interactions. Applying Bader charge analysis, we found that the topmost GL accumulates an extra charge of 0.32 electrons, primarily on atoms positioned directly above the BL atoms. Compared to similar analysis for the BL/SiC system, this excess negative charge on the GL is donated from both the underlying BL and the Si_L_ atoms.

### Adsorption of the F6TCNNQ on the GL(s)/SiC(0001̅) Interface

The adsorption of the F6TCNNQ acceptor was studied on 3 × 3 three-SiC-bilayer reconstructed supercells covered by one and two GLs. For both substrates, the most stable adsorption site is achieved when the benzenoid rings of F6TCNNQ are positioned directly above the C–C bond of the lower GL, as detailed in Supporting Information Figure S5. In this configuration, the minimum distance between periodic images of the adsorbates exceeds 4 Å. In the HSE-optimized models, the adsorbate adopts an almost planar structure with slightly anchored cyano and fluorine ligands, maintaining an average separation of 3.3 Å from the adsorbent. This distance, comparable to the interlayer spacing in graphite, suggests similar weak vdW interactions between the substrate and the molecular F6TCNNQ adsorbate.

Extracting the geometry of the F6TCNNQ molecule from the equilibrium models, we first computed the quasiparticle and adsorption spectra for the freestanding adsorbate, as shown in Figure 7. The *G*_0_ *W*_0_@PBE quasiparticle spectrum indicates a gap of 3.93 eV (IE = 8.48 eV and EA = 4.55 eV) and the corresponding BSE-estimated absorption shows a strong excitation at 2.37 eV (ϵ_b_ = 1.56) attributed to the dominant HOMO-to-LUMO transition. The latter agrees well with the solution absorbance UV–visible spectrum (measured in dichloromethane solvent),^34^ particularly for the first peak at 2.58 eV (481 nm). In Supporting Information Figure S6, we also gathered the quasiparticle and optical spectra obtained using hybrid starting points, as well as those obtained through the ev*GW* scheme, which perfectly align with the available experimental data.

**Figure 7:**
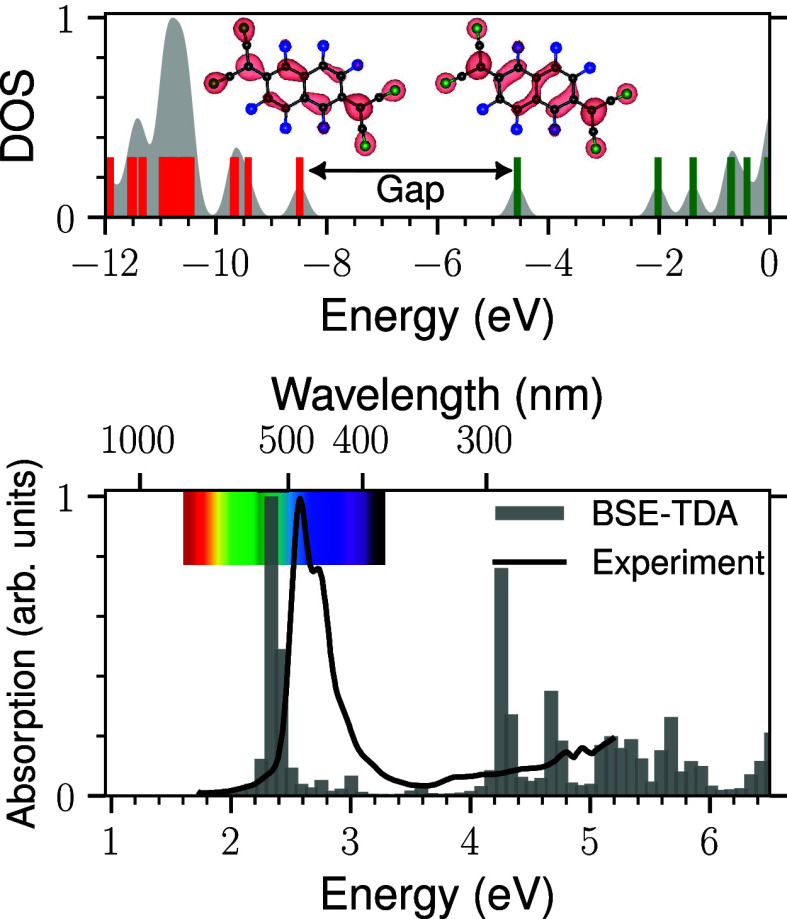
Quasiparticle energies obtained by *G*_0_ *W*_0_@PBE and the corresponding optical absorption lines for the freestanding F6TCNNQ molecule. Occupied and unoccupied states are indicated by red and green vertical bars, respectively, and the spatial distribution of the HOMO and LUMO is illustrated. The experimental absorption data, shown by the black solid curve, is taken from ref 34.

Figure 8a illustrates the electronic band structures of F6TCNNQ/BL/4H-SiC(0001̅), calculated using DFT-HSE and *G*_0_ *W*_0_@PBE approaches. The DFT-HSE results reveal that the splitting and band dispersion of the localized C_D_ states are similar to those of the clean substrate, shown in Figure 4. By projecting the molecular states, we identified the localized LUMO of the adsorbate at 0.45 eV above the VBM, while the HOMO is hybridized with multiple states of BL/SiC, positioned about 1 eV below the VBM (see green lines in the band structure). Consequently, the HSE results indicate that the HOMO–LUMO gap of F6TCNNQ on the substrate is 1.45 eV, which is close to that of the isolated molecule (see the HSE-DOS of the molecule in Supporting Information Figure S5). This surface-independent gap underscores the inability of the HSE functional to account for image charge effects and substrate-induced screening, which can significantly influence adsorbate energy levels.^48^ To capture these effects, we performed *G*_0_ *W*_0_@PBE calculations, as performing *G*_0_ *W*_0_@HSE for such a large system is computationally prohibitive. The quasiparticle correction preserves a qualitatively similar electronic structure, with the splitting between C_D_ states being smaller than that obtained from HSE, yet consistent with the results shown in Figures 2–4. However, upon inspecting the adsorbate’s frontier orbitals in the quasiparticle band structure (Figure 8a), we find that the LUMO is located at 1.4 eV above the VBM, while the HOMO is positioned at −1.1 eV. As a result, the quasiparticle HOMO–LUMO gap of the F6TCNNQ molecule adsorbed on BL/4H-SiC(0001̅) is 2.5 eV, significantly reduced compared to the quasiparticle HOMO–LUMO gap of the isolated molecule in Figure 7, which is 3.93 eV at the same level of theory. This substantial renormalization of the adsorbate gap highlights the critical role of substrate-induced screening effects, which are effectively captured by the *GW* approach.^24,48,49^

**Figure 8:**
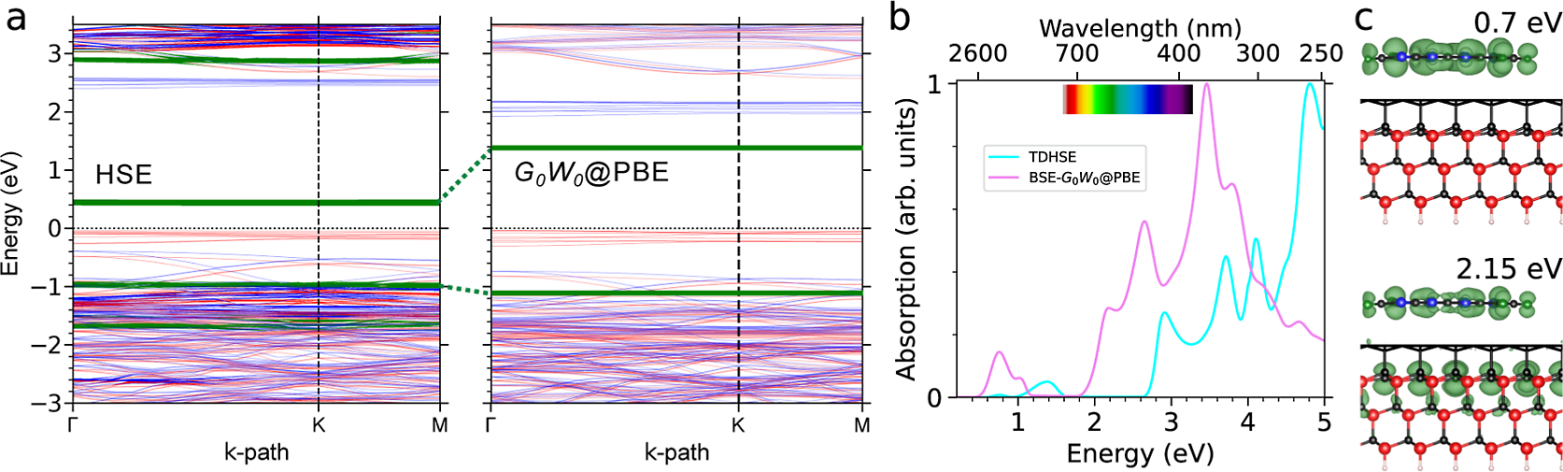
(a) Spin-polarized band structures and (b) the corresponding optical absorption spectra of F6TCNNQ on BL/4H-SiC (0001̅). Projected bands onto the F6TCNNQ states are illustrated by green lines. (c) The spatial distribution of excitons corresponding to transitions at 0.7 and 2.15 eV in the BSE spectrum.

Compared to the absorption spectra of BL/4H-SiC(0001̅) shown in Figure 4, molecular adsorption on this surface introduces a new optical profile, as illustrated in Figure 8b. TDHSE reveals distinct excited states ranging from 1.2 to 1.6 eV (NIR region), with the involved e–h states indicating intermolecular transitions between the hybridized HOMO and the LUMO of the adsorbate. The *G*_0_ *W*_0_-RPA (without e–h interactions) provides a qualitatively similar optical structure within the visible energy range, although with a noticeable blue-shift (see Supporting Information Figure S8). This indeed confirms that the quality of near-edge orbitals, especially those influenced by the adsorbate, is comparable in both the PBE and HSE solutions.

Applying quasiparticle corrections and incorporating bound excitonic effects, the *G*_0_ *W*_0_-BSE spectrum displays well-separated excitations at NIR region. Particularly, transition at 0.75 eV shows a localized Frenkel-like characteristic of the adsorbate LUMO when examining the corresponding exciton wave functions (see Figure 8c). Comparing this intramolecular excitation with the lowest excitations of the isolated F6TCNNQ molecule, falling at 2.37 eV as shown in Figure 7, a pronounced renormalization of the F6TCNNQ-derived optical excitation is evident. Thus, the substrate-induced screening not only strongly renormalizes the energy levels and gap of the F6TCNNQ upon adsorption onto the BL/SiC substrate, but the optical excitations of the adsorbate also undergo significant renormalization. The BSE spectrum further reveals three distinct peaks at 2.15, 2.65, and 3.45 eV. Given the similarity between these excitations and those on the clean BL/4H-SiC surface (see Figure 4), the enhanced intensities can be attributed to the contribution of the adsorbate states. For example, the spatial distribution of the 2.15 eV exciton in Figure 8c highlights the combined contributions of the C_D_ electron states and the LUMO of the adsorbate.

Finally, we examine the qualitative effects of F6TCNNQ adsorption on the 4H-SiC(0001̅) covered by two GLs. Given the semimetallic nature of the substrate, as shown in Figure 6, an accurate quasiparticle description would typically require a *GW* approach with at least partial self-consistency. However, due to the prohibitive computational demands of applying *GW* methods beyond the one-shot approach to such a large supercell, our analysis is restricted to the DFT-HSE ground-state results. Notably, the DFT-HSE approach, which incorporates a fraction of exact exchange, provides a more accurate representation of charge transfer and hybridization in interfaces with complex vdW interactions compared to standard DFT (see DFT-PBE results in Supporting Information Figure S9).

The HSE band structure of the F6TCNNQ/GL/BL/SiC system is shown in Figure 9. The bands projected onto the F6TCNNQ adsorbate, in the absence of appropriate screening effects, exhibit surface-independent energy discretization among molecular energy levels, with a HOMO–LUMO gap comparable to that of the isolated molecule. While the graphene-like bands remain similar to those observed on the clean surface in Figure 6, the most prominent feature upon F6TCNNQ adsorption is a 0.25 eV upward shift in the π–π* bands of the topmost GL, accompanied by a slight splitting, pinned by the adsorbate’s LUMO. This shift of the Dirac point above the Fermi level indicates p-type hybridization and partial charge transfer from the substrate to the adsorbate. The hybridization between the topmost GL and the adsorbate is further illustrated by the spatial distribution of the GL_π_ state and the LUMO of the adsorbate, as shown in Figure 9c,d. Bader charge analysis supports this, revealing a partial charge transfer of 0.37 electrons from the substrate to the F6TCNNQ. Similar findings have been reported for F4TCNQ adsorbed on freestanding graphene, where charge transfer and a partially filled adsorbate LUMO results in an interface dipole, increasing the system’s work function.^20,39,50^

**Figure 9:**
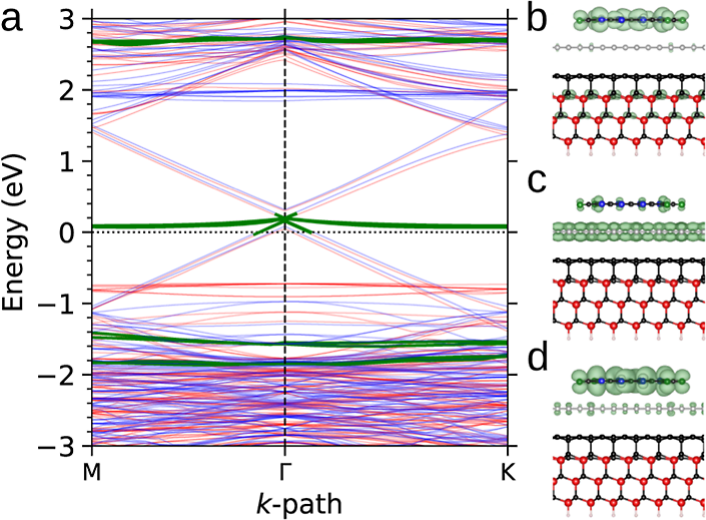
(a) The HSE band structure of F6TCNNQ on GL/BL/4H-SiC (0001̅), with projected molecular states illustrated by green lines. The Fermi energy is set to zero. The isosurfaces of the HSE orbitals for (b) the adsorbate HOMO, (c) the graphene-π band, and (d) the adsorbate LUMO show the degree of the hybridization.

## Conclusion

We performed *GW*/BSE calculations for the two polar faces of 4H-SiC, with and without graphene adlayers. For pristine surfaces, our results indicate that the semiconducting nature of the bulk is retained, albeit with a reduced fundamental gap, resulting in optical absorption within the visible range. The adsorption of the first GL on both polarities, associated with covalent bonding, induces significant changes in the graphene features, with a band gap approximately 2 eV larger on the C-terminated face compared to the Si-terminated face. This difference arises from variations in work function and interfacial interactions, leading to distinct optical absorption characteristics: graphene-covered SiC(0001) exhibits a broader absorption spectrum with an onset in the mid-infrared region, whereas graphene-covered SiC(0001̅) displays an onset in the visible energy range. The adsorption of a second GL restores the characteristic graphene dispersion. However, the position of the Fermi level above the Dirac point indicates n-type doping for graphene on the Si-terminated face, whereas the GL adsorbed on the C-terminated face remains semimetallic.

For the C-terminated 4H-SiC coated with GLs, we also investigated the adsorption of the electron-accepting F6TCNNQ molecule. Our results show that the renormalized LUMO of the adsorbate, positioned within the bandgap of the substrate, leads to the emergence of new optically excited states. These excitations, well-separated from the surface states, offer desirable wavelength selectivity for optoelectronic applications. Additionally, molecular adsorption on the SiC surface coated with two GLs clearly demonstrates p-type doping at the interface, resulting from partial charge transfer from the substrate to the adsorbate.

In conclusion, our comprehensive theoretical study highlights the potential of graphene/SiC interfaces for effectively modulating optoelectronic properties. Additionally, the fine-tuned p- and n-type doping mechanisms at these interfaces offer precise control for various technological applications. From a theoretical perspective, our findings on the optical properties of SiC and Gr/SiC surfaces show close agreement between the TDHSE and BSE-*G*_0_ *W*_0_@HSE results. For molecular adsorption, however, employing a *GW* scheme is crucial for accurately capturing the effects of surface screening on the energy levels of the adsorbate.

## Supplementary Material



## Notes

Additional details on convergence tests, the electronic structure at mean-field levels, geometry optimization, and isolated molecule spectra (PDF)

## References

[1] Levinshtein, M. E. ; Rumyantsev, S. L. ; Shur, M. S. Properties of Advanced Semiconductor Materials: GaN, AIN, InN, BN, SiC, SiGe; John Wiley & Sons, 2001.

[2] Zhao J., Ji P., Li Y., Li R., Zhang K., Tian H., Yu K., Bian B., Hao L., Xiao X. (2024). Ultra-high
mobility semiconducting epitaxial graphene on silicon carbide. Nature.

[3] Ohta T., Bostwick A., Seyller T., Horn K., Rotenberg E. (2006). Controlling
the electronic structure of bilayer graphene. Science.

[4] Ohta T., Bostwick A., McChesney J. L., Seyller T., Horn K., Rotenberg E. (2007). Interlayer
interaction and electronic screening in
multilayer graphene investigated with angle-resolved photoemission
spectroscopy. Phys. Rev. Lett..

[5] Zhou S. Y., Gweon G. H., Fedorov A. V., First P. N., de Heer W. A., Lee D. H., Guinea F., Castro Neto A. H., Lanzara A. (2007). Substrate-induced bandgap opening in epitaxial graphene. Nat. Mater..

[6] Giannazzo F. (2016). Insight into
the mechanisms of chemical doping of graphene on silicon carbide. Nanotechnology.

[7] Hass J., Feng R., Li T., Li X., Zong Z., de Heer W. A., First P. N., Conrad E. H., Jeffrey C. A., Berger C. (2006). Highly ordered graphene for two dimensional
electronics. Appl. Phys. Lett..

[8] Hass J., Feng R., Millán-Otoya J.
E., Li X., Sprinkle M., First P. N., de Heer W. A., Conrad E. H., Berger C. (2007). Structural properties of the multilayer graphene/4H–SiC(0001)
system as determined by surface X-ray diffraction. Phys. Rev. B.

[9] Varchon F., Feng R., Hass J., Li X., Nguyen B. N., Naud C., Mallet P., Veuillen J.-Y., Berger C., Conrad E. H. (2007). Electronic structure
of epitaxial graphene
layers on SiC: effect of the substrate. Phys.
Rev. Lett..

[10] Forbeaux I., Themlin J.-M., Debever J.-M. (1999). High-temperature
graphitization of
the 6H-SiC (0001) face. Surf. Sci..

[11] Berger C., Song Z., Li X., Wu X., Brown N., Naud C., Mayou D., Li T., Hass J., Marchenkov A. N. (2006). Electronic confinement
and coherence in patterned
epitaxial graphene. Science.

[12] Mattausch A., Pankratov O. (2008). Density functional study of graphene overlayers on
SiC. Phys. Status Solidi B.

[13] Coletti C., Riedl C., Lee D. S., Krauss B., Patthey L., von Klitzing K., Smet J. H., Starke U. (2010). Charge neutrality and
band-gap tuning of epitaxial graphene on SiC by molecular doping. Phys. Rev. B.

[14] Ostler M., Deretzis I., Mammadov S., Giannazzo F., Nicotra G., Spinella C., Seyller T., La Magna A. (2013). Direct growth
of quasi-free-standing epitaxial graphene on nonpolar SiC surfaces. Phys. Rev. B.

[15] Daas B. K., Omar S. U., Shetu S., Daniels K. M., Ma S., Sudarshan T. S., Chandrashekhar M. V.
S. (2012). Comparison of epitaxial
graphene growth on polar and nonpolar 6H-SiC faces: on the growth
of multilayer films. Cryst. Growth Des..

[16] Ouerghi A., Marangolo M., Belkhou R., El Moussaoui S., Silly M. G., Eddrief M., Largeau L., Portail M., Fain B., Sirotti F. (2010). Epitaxial graphene on 3C-SiC(111)
pseudosubstrate: structural and electronic properties. Phys. Rev. B.

[17] Darakchieva V., Boosalis A., Zakharov A. A., Hofmann T., Schubert M., Tiwald T. E., Iakimov T., Vasiliauskas R., Yakimova R. (2013). Large-area microfocal spectroscopic
ellipsometry mapping
of thickness and electronic properties of epitaxial graphene on Si-
and C-face of 3C-SiC(111). Appl. Phys. Lett..

[18] Hass J., de Heer W. A., Conrad E. H. (2008). The growth and morphology of epitaxial
multilayer graphene. J. Condens. Matter Phys..

[19] Ruan M., Hu Y., Guo Z., Dong R., Palmer J., Hankinson J., Berger C., de Heer W. A. (2012). Epitaxial graphene on silicon carbide:
introduction to structured graphene. MRS Bull..

[20] Chi M., Zhao Y.-P. (2012). First principle study of the interaction and charge
transfer between graphene and organic molecules. Comput. Mater. Sci..

[21] Rohlfing M., Pollmann J. (2000). Parameter of the Mott-Hubbard
insulator 6HSiC(0001)
√3 × √3R30°: an ab initio calculation. Phys. Rev. Lett..

[22] Cannuccia E., Gali A. (2020). Thermal evolution of
silicon carbide electronic bands. Phys. Rev.
Mater..

[23] Zhang X., Kioupakis E. (2023). Phonon-assisted
optical absorption of SiC polytypes
from first principles. Phys. Rev. B.

[24] Mansouri M., Díaz C., Martín F. (2024). Optoelectronic
properties of electron-acceptor
molecules adsorbed on graphene/silicon carbide interfaces. Commun. Mater..

[25] Hedin L. (1965). New method
for calculating the one-particle Green’s function with application
to the electron-gas problem. Phys. Rev..

[26] Martin, R. M. , Reining, L. , Ceperley, D. M. , Eds. Interacting Electrons: Theory and Computational Approaches; Cambridge University Press, 2016.

[27] Mansouri M., Koval P., Sharifzadeh S., Sánchez-Portal D. (2023). Molecular
doping in the organic semiconductor diindenoperylene: insights from
many-body perturbation theory. J. Phys. Chem.
C.

[28] Heyd J., Scuseria G. E., Ernzerhof M. (2003). Hybrid functionals
based on a screened
Coulomb potential. J. Chem. Phys..

[29] Paier J., Marsman M., Kresse G. (2008). Dielectric properties and excitons
for extended systems from hybrid functionals. Phys. Rev. B.

[30] Yang Z.-h., Sottile F., Ullrich C. A. (2015). Simple
screened exact-exchange approach
for excitonic properties in solids. Phys. Rev.
B.

[31] Mansouri M., Lewis D. K., Sharifzadeh S. (2023). Introduction
of localized spin-state
transitions in the optical absorption spectrum of Cr-doped GaN. Phys. Rev. B.

[32] Otero R., Miranda R., Gallego J. M. (2019). A comparative
computational study
of the adsorption of TCNQ and F4-TCNQ on the coinage metal surfaces. ACS Omega.

[33] de
Oliveira I. S. S., Miwa R. H. (2015). Organic molecules deposited on graphene:
a computational investigation of self-assembly and electronic structure. J. Chem. Phys..

[34] Koech P. K., Padmaperuma A. B., Wang L., Swensen J. S., Polikarpov E., Darsell J. T., Rainbolt J. E., Gaspar D. J. (2010). Synthesis and application
of 1,3,4,5,7,8-hexafluoro tetracyanonaphthoquinodimethane (F6-TNAP):
a conductivity dopant for organic light-emitting devices. Chem. Mater..

[35] Ramsdell L. S., Kohn J. A. (1952). Developments in
silicon carbide research. Acta Crystallogr..

[36] Berger C., Song Z., Li X., Wu X., Brown N., Naud C., Mayou D., Li T., Hass J., Marchenkov A. N. (2006). Electronic confinement
and coherence in patterned
epitaxial graphene. Science.

[37] Cavallucci T., Tozzini V. (2016). Multistable rippling of graphene on SiC: a density
functional theory study. J. Phys. Chem. C.

[38] Zhou Y.-C., Zhang H.-L., Deng W.-Q. (2013). A 3N rule for the
electronic properties
of doped graphene. Nanotechnology.

[39] Pinto H., Jones R., Goss J. P., Briddon P. R. (2009). p-Type doping of
graphene with F4-TCNQ. J. Condens. Matter Phys..

[40] Kresse G., Furthmüller J. (1996). Efficient
iterative schemes for ab initio total-energy
calculations using a plane-wave basis set. Phys.
Rev. B.

[41] Grimme S. (2006). Semiempirical
GGA-type density functional constructed with a long-range dispersion
correction. J. Comput. Chem..

[42] Wang V., Xu N., Liu J.-C., Tang G., Geng W.-T. (2021). VASPKIT: A user-friendly
interface facilitating high-throughput computing and analysis using
VASP code. Comput. Phys. Commun..

[43] Pizzi G., Vitale V., Arita R., Blügel S., Freimuth F., Géranton G., Gibertini M., Gresch D., Johnson C., Koretsune T. (2020). Wannier90 as a community code: new features and applications. J. Phys.: Condens. Matter.

[44] Deslippe J., Samsonidze G., Strubbe D. A., Jain M., Cohen M. L., Louie S. G. (2012). BerkeleyGW:
A massively parallel computer package for
the calculation of the quasiparticle and optical properties of materials
and nanostructures. Comput. Phys. Commun..

[45] Giannozzi P., Baroni S., Bonini N., Calandra M., Car R., Cavazzoni C., Ceresoli D., Chiarotti G. L., Cococcioni M., Dabo I. (2009). QUANTUM ESPRESSO: a
modular and open-source software project for quantum simulations of
materials. J. Phys.: Condens. Matter.

[46] Bruneval F., Rangel T., Hamed S. M., Shao M., Yang C., Neaton J. B. (2016). Molgw 1: many-body
perturbation theory software for
atoms, molecules, and clusters. Comput. Phys.
Commun..

[47] Mansouri M., Casanova D., Koval P., Sánchez-Portal D. (2021). *GW* approximation
for open-shell molecules: a first-principles study. New J. Phys..

[48] Garcia-Lastra J. M., Rostgaard C., Rubio A., Thygesen K. S. (2009). Polarization-induced
renormalization of molecular levels at metallic and semiconducting
surfaces. Phys. Rev. B.

[49] Neaton J. B., Hybertsen M. S., Louie S. G. (2006). Renormalization of molecular electronic
levels at metal-molecule interfaces. Phys. Rev.
Lett..

[50] Chen W., Chen S., Qi D. C., Gao X. Y., Wee A. T. S. (2007). Surface
transfer p-type doping of epitaxial graphene. J. Am. Chem. Soc..

