# Supplementary material for: Tunable Doping and Optoelectronic Modulation in Graphene-Covered 4H-SiC Surfaces
Source: J Phys Chem C Nanomater Interfaces. 2025 Feb 14;129(8):4155–64. doi: 10.1021/acs.jpcc.4c06409 (PMC12282215; doi:10.1021/acs.jpcc.4c06409)
Supplement: Supplementary file 1 [file jp4c06409_si_001.pdf]

Supporting Information

Tunable Doping and Optoelectronic Modulation in Graphene-Covered 4H-SiC Surfaces

Masoud Mansouri[1,*], Fernando Martín[1, 2], and Cristina Díaz[3]

[1]Departamento de Química, Módulo 13, Universidad Autónoma de Madrid, 28049 Madrid, Spain
[2]Instituto Madrileño de Estudios Avanzados en Nanociencia (IMDEA Nano), Campus de Cantoblanco, 28049 Madrid, Spain
[3]Departamento de Química Física, Facultad de CC. Químicas, Universidad Complutense de Madrid, 28040 Madrid, Spain
*masoud.mansouri@uam.es

List of Figures

Figure S1 – Geometry Models 2
Figure S2 – SiC Surface Models 2
Figure S3 – BL/SiC(0001) at the DFT-PBE Level 3
Figure S4 – *GW* Band Structures of GL/BL/SiC 3
Figure S5 – Adsorption Site of the F6TCNNQ 3
Figure S6 – Spectra of Isolated F6TCNNQ 4
Figure S7 – Convergance of Optical Spectra 5
Figure S8 – RPA Spectra for F6TCNNQ/BL/SiC 5
Figure S9 – PBE Results for F6TCNNQ/GL/BL/SiC 6

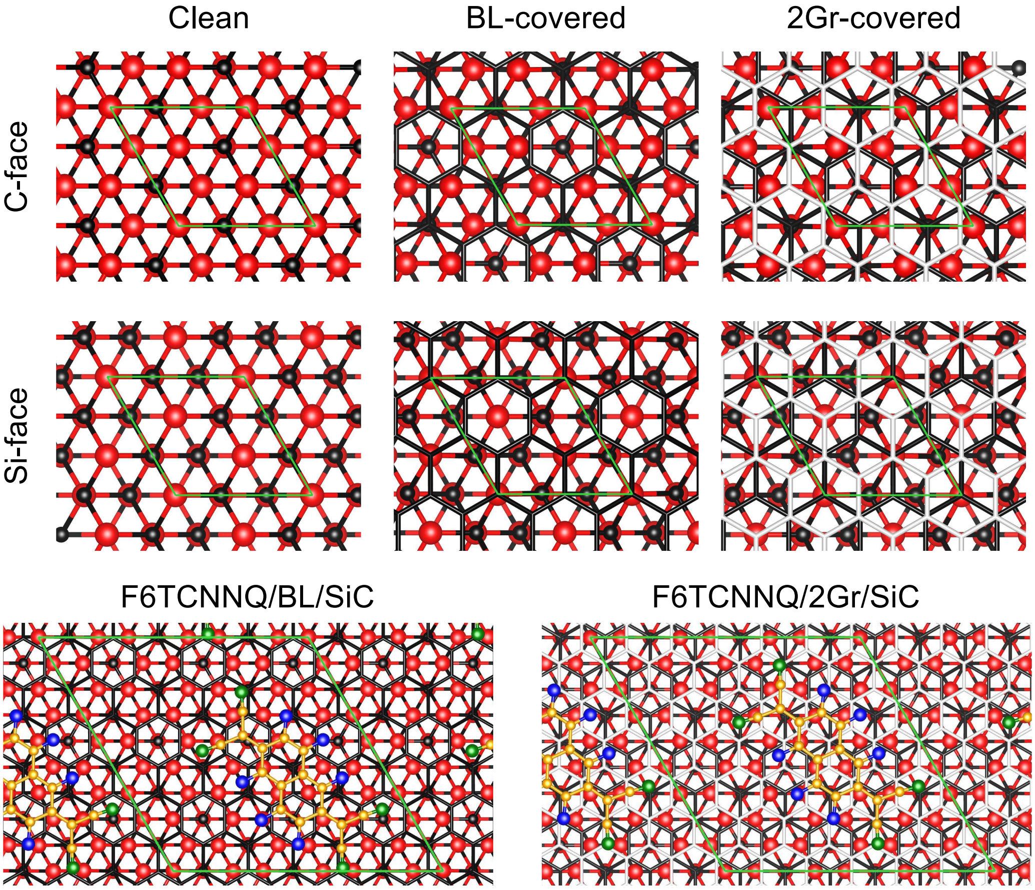

Figure S1: Stick-and-ball model of the studied reconstructed 4H-SiC substrate. BL/GL layers are illustrated with black/silver sticks. The orange, green, and blue circles in the two lowest panels represent the C, F, and N atoms of the adsorbate F6TCNNQ molecule.

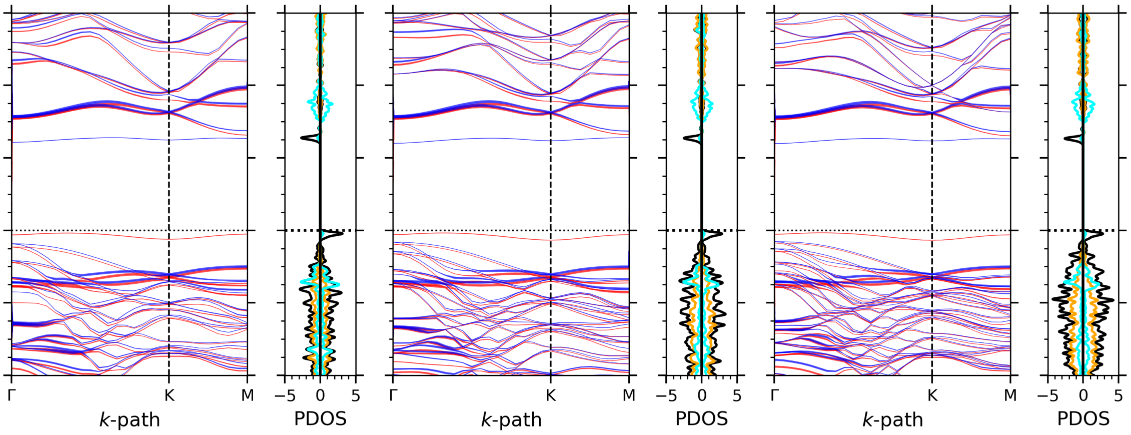

Figure S2: HSE band structures and DOS for BL/4H-SiC $(000\bar{1})$ with (left) three, (middle) four, (right) five SiC-bilayers in the $\sqrt{3}$R supercell. The contribution of the atop BL is given by the thinner lines.

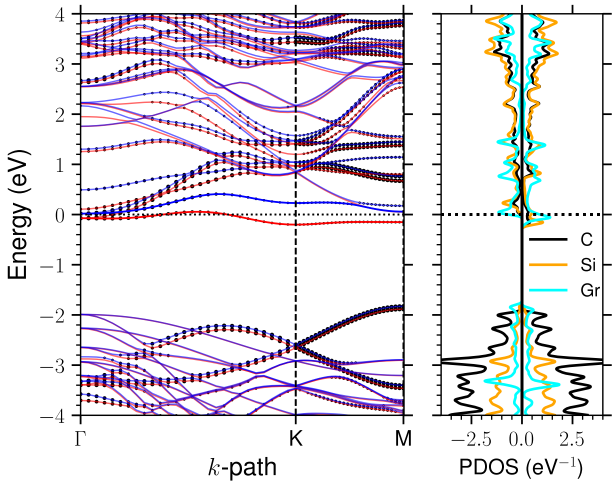

Figure S3: DFT-PBE band structures of BL/4H-SiC(0001), showcasing the metallic ground state.

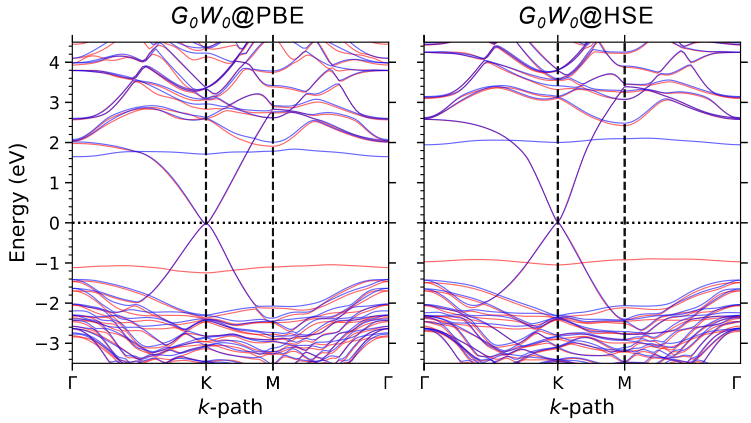

Figure S4: Quasiparticl band structures of GL/BL/4H-SiC(000$\bar{1}$).

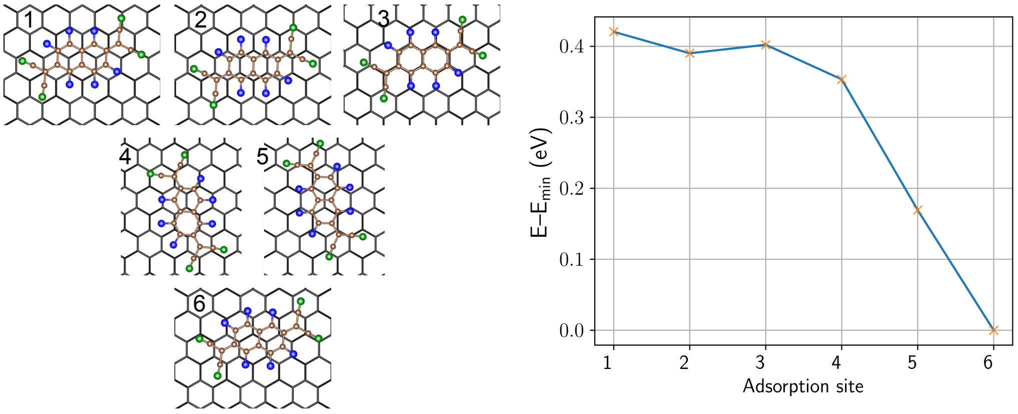

Figure S5: Variation of the total energy of F6TCNNQ on the $\sqrt{3}$R BL/4H-SiC (000$\bar{1}$) surface with respect to the adsorption site.

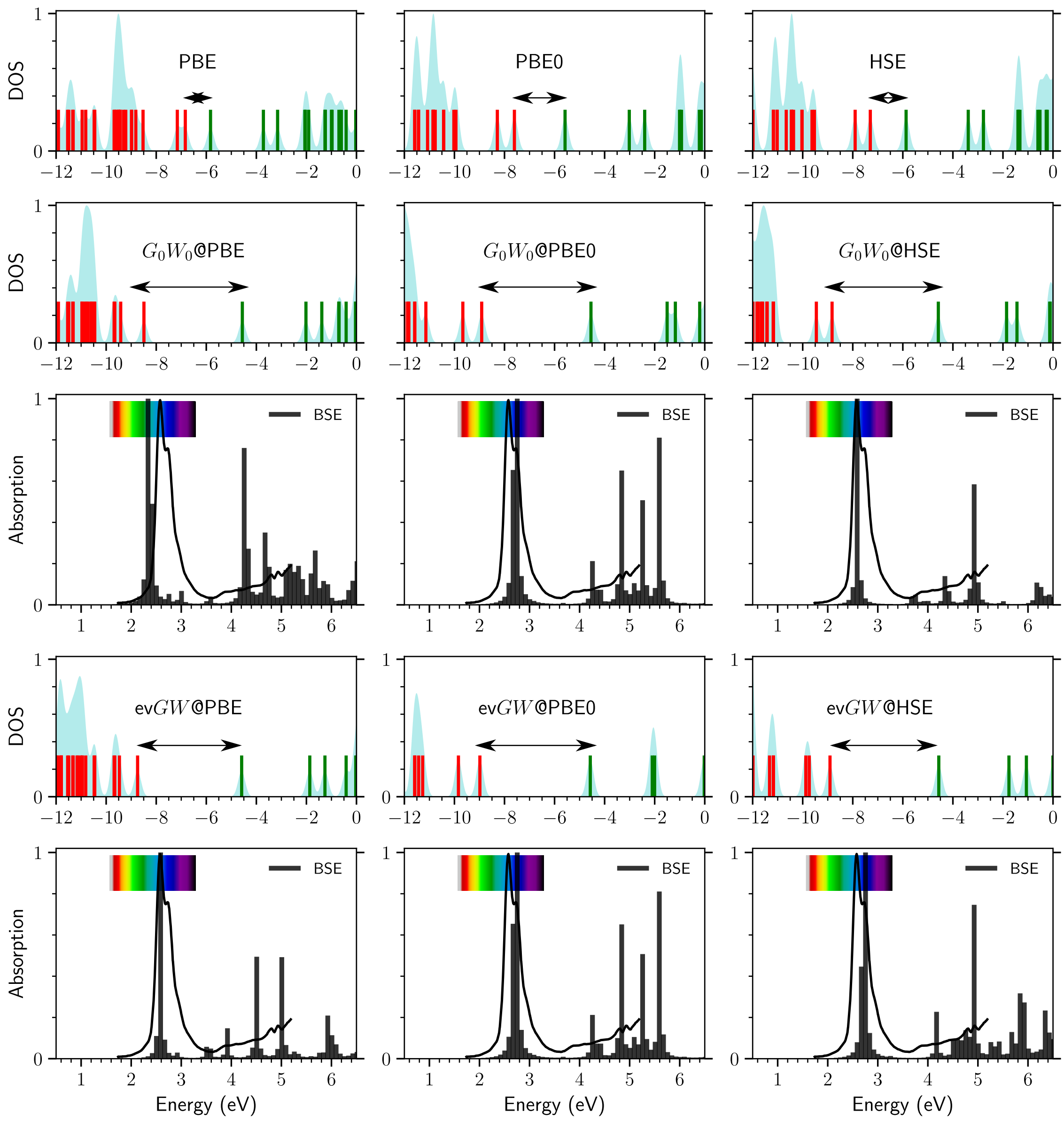

Figure S6: DOS and optical absorption lines of the isolated F6TCNNQ, obtained using different approaches. The black horizontal arrow in DOS shows the HOMO-LUMO gap. BSE spectra in the third and fifth rows were computed using the quasiparticle energies on the second and fourth rows, respectively. The black curve shows the experimental UV-visible absorbance, discussed in the main text.

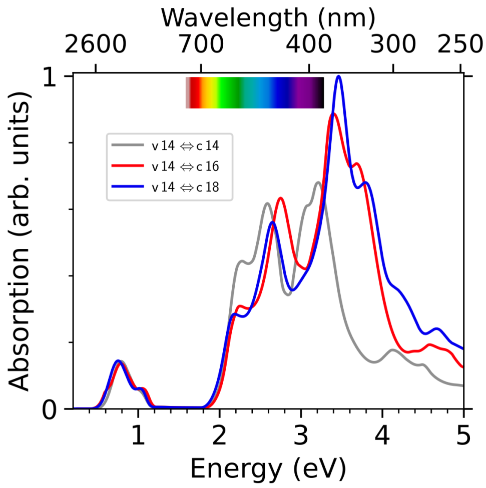

Figure S7: Convergence of the optical absorption spectra with respect to the number of valence and conduction states used in solving the BSE for the F6TCNNQ/BL/4H-SiC system.

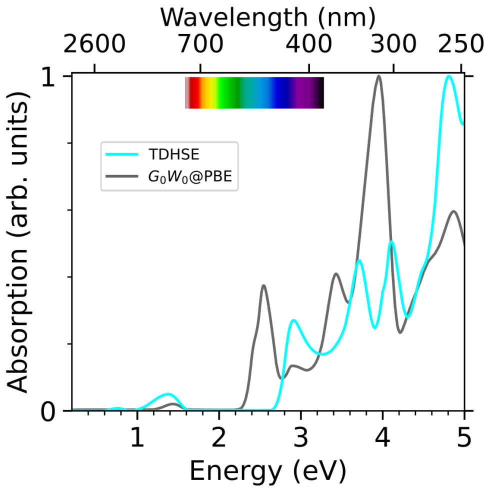

Figure S8: TDHSE and RPA-G_0W_0@PBE absorption spectra for F6TCNNQ/BL/SiC. TDHSE shows weak transitions between C_D and adsorbate LUMO at 0.5 eV, followed by a distinct peak at 1.45 eV, attributed to the intramolecular transitions. RPA-G_0W_0@PBE features qualitatively similar excitations, appearing at 1.4 and 2.5 eV, respectively.

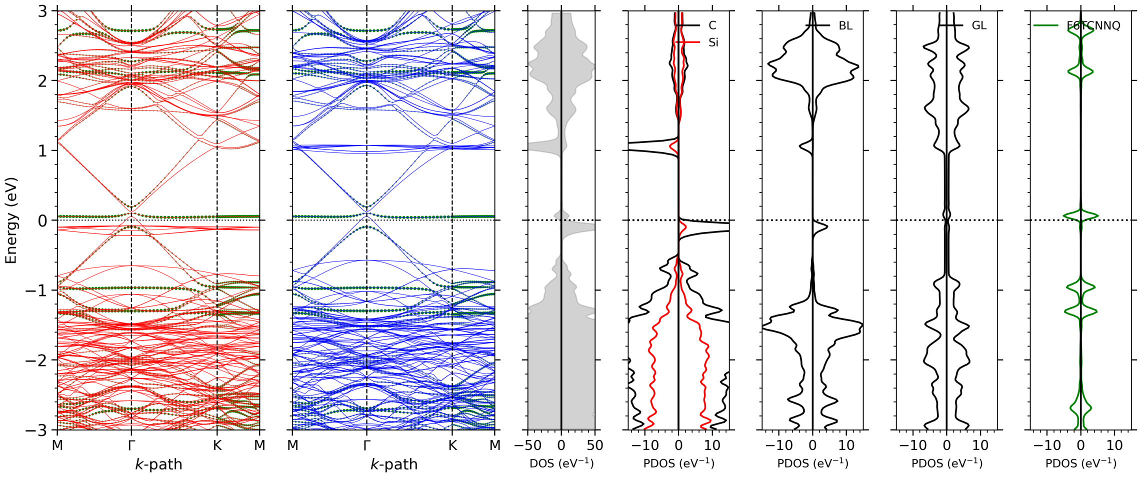

Figure S9: Spin-polarized PBE band structures and PDOS for F6TCNNQ on GL/BL/4H-SiC $(000\bar{1})$. The projected bands on the F6TCNNQ atoms are indicated by enlarged green circles. The Fermi energy is set to zero.
